# Botulinum toxin-type A: could it be an effective treatment option in intractable trigeminal neuralgia?

**DOI:** 10.1186/1129-2377-14-92

**Published:** 2013-11-19

**Authors:** Hatem S Shehata, Mohamed S El-Tamawy, Nevin M Shalaby, Gihan Ramzy

**Affiliations:** 1 Neurology Department, Cairo University, El-Manial 11153, Egypt

## Abstract

**Background:**

Botulinum toxin type A (BTX-A) has been reported to have analgesic effects independent of its action on muscle tone, mostly by acting on neurogenic inflammatory mediators and controlling the neurotransmitter release of sensory and autonomic nerve terminals that are involved in many chronic painful conditions as chronic intractable trigeminal neuralgia (TN).

The aim of our work was evaluating the efficacy, safety, and tolerability of BTX-A for the treatment of intractable idiopathic TN.

**Methods:**

This was a randomized, single-blinded, placebo-control study carried out on 20 Egyptian patients with intractable TN. Patients received a one-time subcutaneous administration of BTX-A using “follow the pain” method. The primary efficacy measure was reduction in pain severity on the 10-cm VAS score as well as in paroxysms frequency from the baseline to week 12 (endpoint last observation carried forward [LOCF]). Secondary efficacy measures included QoL assessment and number of acute medications received from baseline to the endpoint.

**Results:**

Pain reduction at the 12-week endpoint was significant in BTX-A group (p&lt;0.0001); VAS scores at endpoint LOCF relative to baseline for BTX-A group showed a decrease of 6.5 compared with a decrease of 0.3 for placebo, also there was a significant decrease in the number of acute medications and an increase in QoL functioning scale.

**Conclusion:**

These results indicate that BTX-A has a direct analgesic effect in patients with TN and can represent a therapeutic option for intractable cases.

## Background

Trigeminal neuralgia (TN) is defined as a unilateral, abrupt, brief electric shock-like pain, that is limited to the distribution of one or more divisions of the trigeminal nerve [1].

The chronic and recurrent pain imposes a substantial burden on patients’ quality of life (QoL) [2]. Other considerable impacts are resulting from long-term drugs (anticonvulsants) related adverse reactions [3,4]. About 25-50% of patients eventually stop responding to drug therapy and require some form of alternative treatment. Invasive surgical procedures can be reserved to intractable cases [5]. The primary complications of surgery include permanent anesthesia over the face or the troubling dysesthetic syndrome of anesthesia dolorosa-often disabling, is occasionally worse than the original trigeminal neuralgia, and is often untreatable [3], in addition, about half of the patients can have pain recurrence at 2 years [6]. Considering the available therapeutic options, the need for an alternative efficient therapy is overwhelming. The application of the anti-nociceptive effect of botulinum toxin type A (BTX-A) is emerging [7,8].

The analgesic effect of BTX-A has been investigated through a series of open-labeled studies as well as a few randomized controlled trials (RCTs) [9-11], with an increasingly strong evidence that botulinum toxin injections are efficacious, thus placing it as a treatment option either before surgery or for those with intractable TN and unwilling to undergo surgery [5], however, a more placebo-controlled clinical trials are still needed to confirm these findings.

This is a randomized controlled study aiming at evaluating the efficacy and tolerability of botulinum toxin type A (Botox®) for the treatment of intractable idiopathic trigeminal neuralgia.

## Methods

### Study design

This is a randomized, double-blinded, placebo-control study.

### Eligibility and enrollment criteria

Eligible subjects had idiopathic TN according to IHS criteria [1]. In all patients, pain could be elicited by tactile/mild sensory stimulation in different skin areas, consistent with trigger points that affect patients’ QoL, preventing them from shaving, brushing teeth, kissing or washing their faces for fear of triggering pain. We defined intractability as a failure to experience at least a 50% reduction in pain intensity quantified by Visual Analogue Scale (VAS) and/or paroxysms frequency during the last 3 months, despite the use of proper drugs and dosages, prior to study initiation.

### Exclusionary criteria

Patients who responded to medical treatments, pregnants, symptomatic TN (abnormal neurological examination or demonstrable structural lesion by MRI) and patient with possibility of lack of coherence during follow up.

### Protocol approvals and patient consents

The study was designed according to the recommendations of the IHS, and was approved by Neurology Department Review Board in Cairo University. A written informed consent was obtained from all participants prior to study commencement.

### Recruitment, randomization and study phases

From March, 2010 to June, 2012, 20 consecutive Egyptian patients who fulfilled inclusion criteria were recruited.

### Screening phase

**Figure 1:**
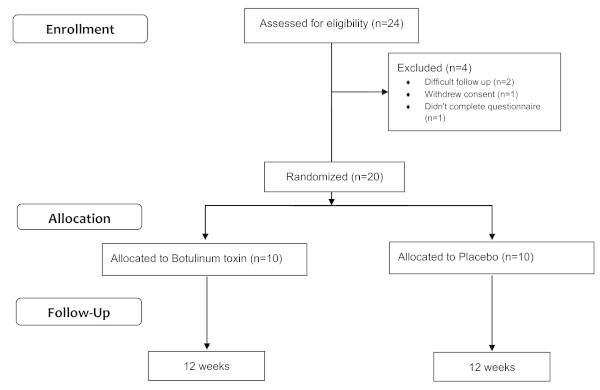
Study flow chart.

Every patient was assessed clinically and by MRI brain. The MRI scans of all patients were normal as a prerequisite for inclusion even for those patients who were subjected to surgery for their idiopathic neuralgias. Thereafter, all patients were requested to complete a baseline, pre-treatment questionnaire to assess pain intensity using VAS, average paroxysms frequency per day, precipitants, associated symptoms, trigger areas, drug compliance, and number of weekly acute medications in the last 3 months. Initially, 24 patients were recruited; 2 of them were excluded as they had difficulty in complying the follow up visits (they were from upper Egypt), one patient withdrew his informed consent and one patient failed to complete the screening questionnaire (Figure 1). After the screening questionnaire, patients were subjected to the 10-point quality of life (QoL) scale adopted from American Chronic Pain Association (Arabic version) [12] ranging from 10 (normal daily activities) and 0 (non-functioning). All patients had been treated with drugs known to improve trigeminal neuralgia, 11 patients were on carbamazepine (600 to 1400 mg) either alone (n = 2), in combination with gabapentine (400 to 1200 mg) (n = 5) or on triple therapy with baclofen (30 to 75 mg) (n = 4). Nine patients were on oxcarbazepine (900 to 1800 mg) either alone (n = 2) or in combination with gabapentine (n = 5). This regimen had been maintained for at least 3 months and was kept unchanged throughout the study period. Two patients (10%) had undergone previous surgical treatment.

### Randomization phase

Every patient received either BTX- A (Botox®) (100 U Botox in 2 mL preservative-free normal saline, resulting in a concentration of 5 units/0.1 mL) or placebo (2 mL 0.9% NaCl). Eligible patients were allocated to one of the two treatment arms in a randomized (1:1), double-blind fashion using a computer-generated randomization to receive either arm with 12 weeks follow up period.

### Injection techniques

Injection points were detected using “follow the pain” method, especially in the trigger zones and each point had received 5 units of BTX-A / or 0.1 ml placebo subcutaneously. In patients with mandibular root involvement, a larger dose of the toxin was injected posteriorly in the masseter to avoid undesired cosmetic effects. Patients were evaluated every 2 weeks for 12 weeks by using the VAS. The number of painful paroxysms and the need of any acute medications were reported.

### Blinding

Assignment to either Botox or placebo was blinded to participants, treating physicians (injectors) and investigators who assessed the patients in follow up visits. Every effort was made to maintain strict blinding; the only unblinded physician (G.R) was the one who performed randomization and prepared the syringes in the injection session. He was trained to perform Botox preparation for injection and he was not involved in any other study procedures. It is worth noting that, this blindness was not maintained after the first 2 weeks assessment in some patients who developed facial asymmetry following BTX-A injection, however, the follow up assessment scales were presented in a structured interview that minimized biased investigator’s impression.

### Efficacy measures

The primary efficacy measures were pain severity reduction in the 10-cm VAS score and paroxysms frequency from the baseline to week 12 (endpoint last observation carried forward [LOCF]). Secondary efficacy measures included QoL scale and number of weekly acute medications received from baseline to the endpoint.

### Safety measures

Any adverse event that a subject reported during the study was recorded by the investigators, graded for severity (mild, moderate, or severe), and assessed for its relationship to study treatment (none, possible, probable, or definite). A serious adverse event was defined as one that was fatal, life-threatening, permanently disabling, or required admission to hospital.

### Data analysis

Data management was carried out with the Statistical Package for Social Sciences (version 12, SPSS Inc. Chicago, IL, USA). Simple descriptive analysis in the form of means and standard deviations were calculated for numerical data, qualitative data were described using percent distribution. Efficacy and safety measures were assessed for all patients based on intention to treat. Comparison of Botox and placebo groups in efficacy measures at endpoints using the LOCF was conducted using an analysis of covariance (ANCOVA) model. Correlation was conducted using bivariate Pearson correlation coefficient. The level of significance was set at 0.05.

## Results

### Participant characteristics

A total of 20 Egyptian patients were randomized. Their age ranged from 27 to 72 years (mean age: 45.95 ± 10.02 years). They were 9 males (45%) and 11 females (55%), the mean duration of the disease was 5.33 ± 1.52 years (range from 3 to 9 years). Pain affected one root in 8 patients, while two roots were affected in 12 patients (Table 1).

**Table 1:** Demographic and clinical data

**Patient**	**Age (years)**	**Gender**	**Duration**	**Branch**	**Trigger points**
**1**	72	M	7	Right V2	Infra orbital
**2**	35	F	5	Left V1	Eye brow
**3**	44	M	4.5	Left V2 & 3	Infra orbital & upper lip & mentalis
**4**	50	M	7	Right V1 & 2	Eye brow & infra orbital
**5**	52	F	9	Right V3	Mentalis
**6**	45	F	6	Right V2 & 3	Infra orbital & upper lip & mentalis
**7**	46	F	5.5	Left V2 & 3	Nostril & upper lip
**8**	32	M	4	Left V1 & 2	Eye brow & infra orbital
**9**	27	F	4.5	Right V2	Upper lip
**10**	48	M	5	Left V2 & 3	Nostril & upper lip
**11**	39	F	6.5	Left V1 & 2	Nostril & mouth angle
**12**	51	F	7.5	Right V3	Mentalis
**13**	39	M	4	Left V3	Mentalis
**14**	42	F	5.5	Left V2 & 3	Infra orbital & upper lip & mentalis
**15**	36	M	3.5	Right V2 & 3	Infra orbital & upper lip & mentalis
**16**	47	F	3	Left V 3	Mentalis
**17**	55	M	4	Left V2 & 3	Infra orbital & upper lip & mentalis
**18**	49	F	5	Right V 3	Mentalis
**19**	56	M	4	Left V2 & 3	Infra orbital & upper lip & mentalis
**20**	54	F	6	Left V2 & 3	Infra orbital & upper lip & mentalis

M: male, F: female, V1: first division of trigeminal N (ophthalmic), V2: second division of trigeminal N (maxillary), V3: third division of trigeminal nerve (mandibular).

### Injection paradigm

Overall, the enrolled 20 patients completed the 12-week study period after the injection session, of 10 patients receiving the subcutaneous botulinum toxin injection (BTX-A group). The overall injected doses ranged from 40 units (8 injection points) to 60 units (12 points) (mean ± SD was 48 ± 5.87).

### Efficacy variables

#### Primary measures

**Figure 2:**
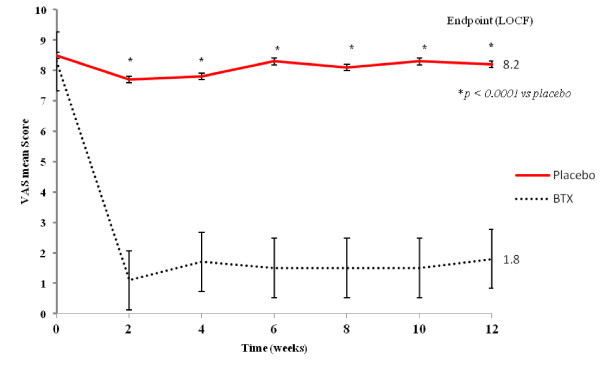
Mean scores of VAS during follow up visits.

The mean baseline pain scores in placebo group and BTX-A group were 8.5 and 8.3 respectively on the VAS score with no significant difference. Pain reduction at the 12-week endpoint was significant in BTX group (P < 0.0001), VAS scores at endpoint LOCF relative to baseline for BTX-A group showed a decrease of 6.5 compared with a decrease of 0.3 for placebo. This significant reduction in the mean VAS scores were recorded in BTX-A group in week 2 and maintained over the follow up visits (Figure 2). The paroxysms frequency reduction was significantly noted in BTX-A group from week 2 and continued till the endpoint LOCF compared to placebo group (P < 0.0001) (Table 2).

**Table 2:** Efficacy variables in the studied groups

		**BTX-A**		**Placebo**		**P value**
		**Mean**	**SD**	**Mean**	**SD**	
Paroxysm frequency /day	Baseline	36.7	3.13	39.20	3.05	0.087
	Endpoint	4.0	7.12	36.10	13.45	0.0001
Number of weekly acute medications	Baseline	32	2.11	30.9	1.79	0.225
	Endpoint	4.7	2.31	30.3	1.77	0.0001
QoL scale	Baseline	3.10	0.88	3.10	1.10	1.0
	Endpoint	9.0	1.49	3.40	0.97	0.0001

#### Secondary measures

There was a significant decrease in number of weekly acute medications and increase in QoL functioning scale with (Table 2).

### Dose efficacy relationship

There was no significant correlation between the total injected dosages of BTX-A and endpoint VAS (r = 0.327, P = 0.356) or paroxysms frequency (r = −0.484, P = 0.156).

### Adverse events

Treatments were generally tolerated, no systemic reactions were noted and there were no serious injections-related adverse events. The reported side effects were facial asymmetry, hematoma at the site of injection, itching and pain at the site of injection (Table 3). All these side effects were transitory and did not interfere with the patient activity, and did not need further management.

**Table 3:** Detected adverse events

	**BTX-A**	**Placebo**
Facial asymmetry	4	0
Hematoma at site of injection	1	2
Itching at site of injection	1	1
Pain at site of injection	1	1

## Discussion

The role of BTX-A in the treatment of drug-refractory trigeminal neuralgia has been evaluated by some authorities [9,13,14], and it was found to be an effective treatment with the majority of the patients reporting a reduction or even disappearance of the pain. BTX-A was found to be effective in combination with pharmacotherapy, prior to considering more invasive therapies such as surgery or gamma knife radiosurgery. As such, BTX-A is a particularly valuable treatment for elderly patients and those with adverse anesthetic comorbidities [8,15]. However, these studies included small patients number or of low-quality RCTs [16].

The mechanism by which BTX-A exerts its antinociceptive effect is poorly understood; whether or not BTX-A can change sensory perception in those patients remains unknown [17]. Cui et al [18]. demonstrated that subcutaneous BTX-A injection is associated with the inhibition of formalin-induced glutamate release; an important mediator for the induction and maintenance of central sensitization of pain [19]. BTX-A may also reduce the peripheral nociceptive input by inhibiting the release of substance P and calcitonin-gene-related peptide (CGRP), both of which would play a very important role in neurogenic inflammation [20-22]. Moreover, BTX-A can reduce the release of other neurotransmitters and neuromediators including epinephrine and norepinephrine [23]. The effect of BTX-A has also an indirect central action through centrally mediated signal transduction to the spinal trigeminal nucleus [24,25]. Another direct effects on muscle nociceptors and alteration of afferent derived from muscle spindle might play a role according to Arezzo [26] especially in reducing the myofascial pain by inhibiting muscle spasms-pain cycle.

The results of the current study support the possible role of BTX-A in pain alleviation, evidenced by the significant reduction in the mean VAS scores as well as the paroxysms frequency in BTX group in week 2 which continued till the endpoint LOCF, in addition to the significant increase in QoL functioning scale compared to the placebo group. Similar to our data, Pioversan et al. [27] reported significant pain reduction in 13 patients with trigeminal neuralgia within a period of 10 days; while in 20 days, patients did not exhibit any obvious symptoms. Some other studies reported a significant effect within 1–2 weeks with a maximum effect within 4–6 weeks [9,14]. The long-term efficacy and its stability of its effectiveness of BTX-A in TN was elaborated in two studies [13,28] which suggested that the effect of a single BTX-A injection could last for 6 mo (24 weeks), whereas other studies showed waning of efficacy started from the 8th week after treatment [28].

In our series a mean of 5 units /cm^2^ was injected with a mean total dose of 48 units. In Pioversan et al. [27] study, the Mean BTX-A dose was 3.22 units/cm^2^. An open label study assessed the clinical effects of BTX-A injections in 12 patients with otherwise unresponsive idiopathic trigeminal neuralgia. Patients were infiltrated with 20–50 units of BTX-A in trigger zones. The patients were assessed on a weekly basis using the Visual Analogic Scale for pain. Ten patients reported a significant benefit from BTX-A injections, with reduction or even disappearance of pain, and remained pain free for as long as 60 days [14]. In another open-ended study to investigate, 8 patients with refractory trigeminal neuralgia were administered 100 U BTX-A through an injection into the zygomatic arch. The study concluded that BTX-A was quite effective in treating trigeminal neuralgia without the risk of excessive side effects [9]. In our assessment of total BTX-A dosages used and drug efficacy measures, we could not find a significant correlation, which could be attributed to the fixed dose per site regimen adopted in our series, thus further evaluation of using different dosages per site can be a point of investigation.

Regarding safety of the BTX-A in the current study, side effects were generally tolerated and were mostly related to the injection sites, 4 cases developed mild facial asymmetry which was self limited and did not impose much impact on the patients especially in the setting of reduced pain. However, this adverse event could be overcome by a more posterior injection approach.

## Conclusion

Subcutaneous BTX-A injection can result in relieving pain in intractable cases with TN with minimal adverse reactions and can be an effective treatment option.

## Competing interests

The authors declare that they have no competing interests.

## Authors’ contribution

HS, MEl-T, and NS participated in the pre-injection assessment and in injection as a team, whereas, GR participated in post-injection assessment. All authors read and approved the final manuscript.
